# Automated Optimized Synthesis of [^18^F]FLT Using Non-Basic Phase-Transfer Catalyst with Reduced Precursor Amount

**DOI:** 10.3390/molecules27238323

**Published:** 2022-11-29

**Authors:** Olga S. Fedorova, Viktoriya V. Orlovskaya, Raisa N. Krasikova

**Affiliations:** N.P. Bechtereva Institute of the Human Brain, Russian Academy of Sciences, 197376 St. Petersburg, Russia

**Keywords:** fluorine-18, radiofluorination, automated synthesis, 3′-deoxy-3′-[^18^F]fluorothymidine, [^18^F]FLT, solid phase extraction

## Abstract

3′-deoxy-3′-[^18^F]fluorothymidine ([^18^F]FLT) is a positron emission tomography (PET) tracer useful for tumor proliferation assessment for a number of cancers, particularly in the cases of brain, lung, and breast tumors. At present [^18^F], FLT is commonly prepared by means of the nucleophilic radiofluorination of 3-*N*-Boc-5′-*O*-DMT-3′-*O*-nosyl thymidine precursor in the presence of a phase-transfer catalyst, followed by an acidic hydrolysis. To achieve high radiochemical yield, relatively large amounts of precursor (20–40 mg) are commonly used, leading to difficulties during purification steps, especially if a solid-phase extraction (SPE) approach is attempted. The present study describes an efficient method for [^18^F]FLT synthesis, employing tetrabutyl ammonium tosylate as a non-basic phase-transfer catalyst, with a greatly reduced amount of precursor employed. With a reduction of the precursor amount contributing to lower amounts of synthesis by-products in the reaction mixture, an SPE purification procedure using only two commercially available cartridges—OASIS HLB 6cc and Sep-Pak Alumina N Plus Light—has been developed for use on the GE TRACERlab FX N Pro synthesis module. [^18^F]FLT was obtained in radiochemical yield of 16 ± 2% (decay-corrected) and radiochemical purity >99% with synthesis time not exceeding 55 min. The product was formulated in 16 mL of normal saline with 5% ethanol (*v*/*v*). The amounts of chemical impurities and residual solvents were within the limits established by European Pharmacopoeia. The procedure described compares favorably with previously reported methods due to simplified automation, cheaper and more accessible consumables, and a significant reduction in the consumption of an expensive precursor.

## 1. Introduction

In the field of molecular imaging, positron emission tomography (PET) is seen as a leading approach for the visualization and quantification of biochemical processes in living subjects. The use of biologically active molecules labelled with short-lived positron-emitting radionuclides (PET radiotracers) enables better understanding of disease pathways, evaluate effects of new therapeutic agents, monitors disease progression and treatment responses, as well as measures the pharmacokinetics and pharmacodynamics of drug candidates. In combination with computed tomography, PET-CT is currently the main tool in oncology clinical diagnostics. Of the cyclotron-produced PET radionuclides, fluorine-18 is the most frequently used due to the combination of a low positron energy (0.635 MeV) and relatively long half-life (109.8 min), allowing for complex synthetic approaches, and providing access to a variety of fluorine-18 labeled radiopharmaceuticals.

**Figure 1:**
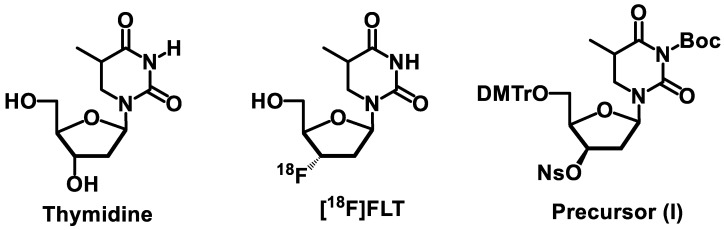
The structures of thymidine, [^18^F]FLT and labeling precursor, 3-*N*-Boc-5′-*O*-dimethoxytrityl-3′-*O*-nosyl-thymidine (**I**).

Of these, alongside the [^18^F]FDG, 3′-deoxy-3′-[^18^F]fluorothymidine ([^18^F]FLT, Figure 1), a labelled fluorinated thymidine analogue, introduced in 1998 [1], has been one of the most utilized in the clinical PET oncology imaging to date [2]. Interacting with cytosolic thymidine kinase (TK1) primarily expressed in the dividing cells during the S-phase of the DNA synthesis, [^18^F]FLT acts as an effective quantitative marker for cell proliferation [3]. The results obtained with [^18^F]FLT PET are in good correlation with the measurements of Ki-67, the gold standard histological marker of proliferation [4]. Numerous studies confirm that [^18^F]FLT uptake reflects tumor proliferation in most human cancers, especially for brain, lung and breast tumors [5,6,7,8,9]. This radiolabeled nucleoside has been particularly useful as a prognostic tool for the treatment response in standard chemotherapy and radiotherapy [10] as well as the early assessment of the therapy response in cases of new therapeutics [11].

At present, [^18^F]FLT is commonly prepared in a two-step one-pot synthesis through nucleophilic aliphatic fluorination (S_N_2) using a suitable thymidine-based precursor, followed by the removal of the protecting groups. Since the initial synthesis [1], various precursors for [^18^F]FLT have been proposed and evaluated in manual and automated radiolabeling procedures. A comparison of selected precursors and their radiochemical yields has been covered in a relatively recent review [12]. Among the precursors, 3-*N*-Boc-5′-*O*-dimethoxytrityl-3′-*O*-nosyl-thymidine (**I**, Figure 1) [13,14], is, to date, the most used one and is commercially available in GMP grade.

Nucleophilic displacement of a 4-nitrobenzenesulfonyl (nosyl, ONs) leaving group in **I** with [^18^F]fluoride usually is performed in the presence of a kryptofix/carbonate (K2.2.2/K_2_CO_3_) complex [15] or tetrabutylammonium hydrocarbonate (TBAHCO_3_) [16] as phase transfer catalysts (PTC) in acetonitrile. To ensure high radiochemical yields, large quantities of the precursor (20–40 mg, 24–48 μmol) are commonly used, resulting in the formation of substantial amounts of chemical impurities. Despite relatively high radiochemical yields (40–65%, decay corrected) [15], the necessity of a lengthy semi-preparative HPLC procedure for purification, even in cassette-based automated modules [15], make these methods less attractive for the routine clinical production of [^18^F]FLT. The use of tertiary alcohols, such as *t*-butanol [17,18] or 3-methyl-pentan-3-ol [19] as co-solvents for radiofluorinations, was found to be effective in improving radiochemical conversion (RCC) and activity yield with a relatively low amount of precursor used. However, for various practical reasons these solvents are not seen as ideal for the clinical production of radiotracers.

As an alternative to HPLC purification, the development of solid-phase extraction (SPE) techniques based on commercially available disposable cartridges has been the focus of radiopharmaceutical research for many radiotracers including [^18^F]FLT [16,20,21,22]. The SPE method, in principle, is very simple, fast, reliable, and is easily adaptable to most automated equipment. However, in contrast to the HPLC, to achieve a satisfactory chemical purity of the radiotracer through cartridge-based purification is a challenging task, especially considering the formation of potentially toxic side-products such as 2′,3′-didehydro-2′,3′-dideoxythymidine (stavudine) [23]. To date, several SPE purification procedures for the [^18^F] FLT have been described [16,20,21,22]. With 20–25 mg of **I** typically used, removal of chemical impurities was achieved by means of combining different type of SPE cartridges and intermediate sorption/eluting steps, this, in turn, required more complex automation.

Previously, we have described a method for achieving efficient aliphatic radiofluorinations using tetrabutylammonium tosylate (TBAOTs) as non-basic PTC while minimizing amounts of the labeling precursors used [24,25,26]. According to the preliminary data [26], TBAOTs-mediated fluorination of **I** provided around 70% RCC with only 1–2 mg of precursor used. As such, this approach was worth considering for the automated production of [^18^F]FLT. Here were report an automated synthesis of [^18^F]FLT with a fast and straightforward purification protocol that, due to considerable reduction in reagent amounts, required only two disposable cartridges. With this procedure implemented in a TRACERlab FX N Pro (one of the most widely used automation platforms) sufficient quantities of [^18^F]FLT at high radiochemical and chemical purity could be reproducibly generated.

## 2. Results and Discussion

### 2.1. Optimization of the [^18^F]FLT Radiosynthesis under Remote-Controlled Operation

**Scheme 1:**
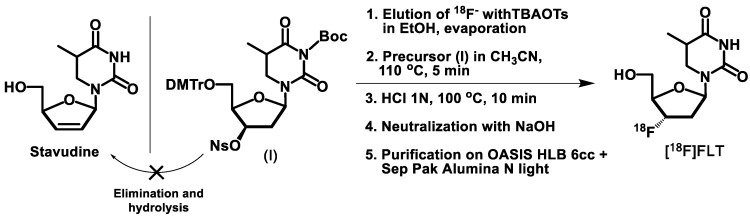
Radiosynthesis of [^18^F]FLT and route to the formation of 2′,3′-didehydro-2′,3′-dideoxythymidine (stavudine).

[^18^F]FLT was produced as outlined in Scheme 1.

Based on our previous findings [24,25,26], TBAOTs-mediated radiosynthesis of the [^18^F]FLT was taken as the starting point for implementation into an automated production. To begin, the radiosynthesis was performed on a remotely-controlled apparatus (in-house development). The reactions were carried out in 5 mL V-vials with a screw-cap (Wheaton) placed in a heating block. All of the reagents transfer was achieved through nitrogen flow. The module used is described elsewhere [27]. In brief, [^18^F]fluoride was trapped on the QMA cartridge (130 mg) and radioactivity was eluted backwards relative to the loading direction using a solution of TBAOTs in 0.8 mL of MeOH, with the eluate collected directly into the reaction vessel. With this approach the solvent was removed in a single step, by heating under gas flow, eliminating the traditional azeotropic drying procedure. Initially the radiofluorination step was carried out in acetonitrile (110 °C, 5 min) using 1.1 µmol of **I** and 2.5 µmol of TBAOTs, as described previously [26]. However, lower and somewhat variable ^18^F-incorporation rates (RCCs 43–52%) were observed. This discrepancy may be attributed to the differences in the quality of the precursor from different commercial sources. Complaints regarding the quality of the commercially-available starting material for the [^18^F]FLT have been reported by others [16].

**Figure 2:**
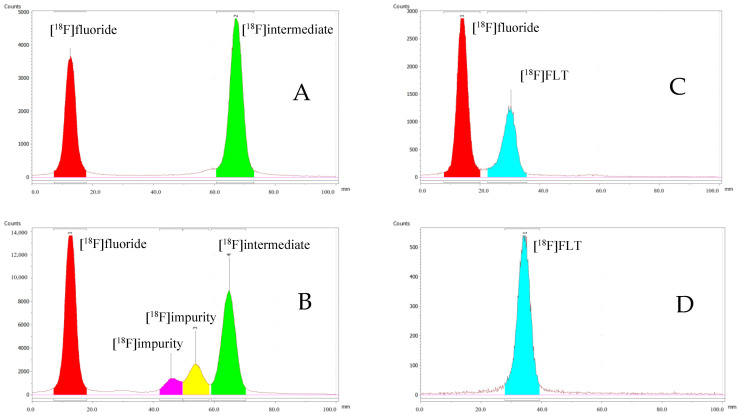
RadioTLC data for the synthesis of [^18^F]FLT; eluent—ethyl acetate. (**A**) remote-controlled synthesis; reaction mixture after radiofluorination; 2.4 µmol, of **I**, 4.8 µmol of TBAOTs, 110 °C. 5 min. RCC 62%; (**B**) remote-controlled synthesis; reaction mixture after radiofluorination; 4.8 µmol, of **I**, 9.6 µmol of TBAOTs, 110 °C, 20 min; (**C**) automated synthesis; reaction mixture after hydrolysis; 2.4 µmol of **I**, 4.8 µmol of TBAOTs; the content of the [^18^F]FLT in the reaction mixture 32%; (**D**) automated synthesis; [^18^F]FLT, final formulation.

The results have prompted us to re-investigate the radiofluorination conditions using different amounts of precursor and variable temperatures. With 4 mg (4.8 µmol) of **I** and an equimolar amount of TBAOTs, up to 50% RCC was obtained in 5 min radiofluorination at 110 °C. Increasing the reaction time to 10 and then to 20 min led to the formation of unidentified labelled by-products (Figure 2B; Table S1). In a series of follow-up experiments, the effect of the molar ratios of PTC/precursor on the radiolabeling efficiency has been evaluated. It was found that the double excess of the PTC over precursor (4.8 µmol/2.4 µmol) provided an RCC of 60–75% (Figure 2A). These amounts of reagents and the reaction time of 5 min have been judged to be optimal and were accepted for the automation procedures.

According to the literature data, only one example of a similarly low-quantity of **I** (5 mg) in a kryptofix-mediated fluorination has been reported to date [19]. The reaction was carried out in 800 μL of acetonitrile and 750 μL of 3-methyl-pentan-3-ol (110 °C, 15 min) with substantially reduced quantity of base (12–15 mg of K222, 0.59 mg of K_2_CO_3_) providing RCC as high as 93%. After acid hydrolysis, [^18^F]FLT was recovered from the crude reaction mixture by semi-preparative HPLC with a decay corrected RCY of 54% in 80 min of synthesis time. The authors [19] did not provide the data either on the residual amounts of the reaction solvent in the final preparation, or any information on its permissible levels. Therefore we have found acetonitrile to be a more appropriate reaction solvent, for which the allowable limits are well-established.

The next step was the hydrolysis of the Boc and DMTr protective groups in ^18^F-fluorinated intermediate using 1.0 mL of 1 N HCl, which was then neutralized with 2.6 mL of 0.3 N NaOH in 10 mL of water. At this stage, the deprotection step was performed as described in the literature, at 100 °C for 10 min, resulting in a quantitative deprotection (Figure 2C, peak of the ^18^F-fluorinated intermediate was not detected).

As it was already mentioned, the development of SPE purification procedure providing sufficient radiochemical and chemical purity of the product was one of the more challenging tasks in the whole synthesis procedure. Under optimal conditions the only radiochemical impurity was the unreacted [^18^F]fluoride, removed by means of a suitable reverse-phase cartridge. The residual amounts of [^18^F]F^-^ are removed on the Alumina N cartridge that is commonly used in the last stage of SPE purification of [^18^F]FDG and many other radiotracers. As for chemical impurities in the [^18^F]FLT preparation, care must be taken over the presence of 2′,3′-didehydro-2′,3′-dideoxythymidine (stavudine) formed in the competing elimination reaction during the first synthesis step (Scheme 1). This compound is a potentially toxic antiretroviral drug with the release criteria for [^18^F]FLT of 0.1 mg/V, where V is a maximum recommended dose in milliliters [23]. Other unlabeled impurities of concern are thymine and thymidine, subject to the same release criteria of 0.1 mg/V [23]. As seen in Table 1, despite similar amounts of the precursor used, the content of these three impurities has varied greatly between different manufacturers, depending on the type of the SPE cartridges used and their combination.

Based on our findings when it comes to using lower amounts of the precursor, we aimed to develop a simplified SPE purification procedure with a minimal number of cartridges and without the intermediate steps. After screening several cartridges, we ended up choosing HLB, a multi-purpose reversed-phase sorbent that provides high retention capacity for a wide range of compounds, having no silanol groups, and being capable of retaining basic, acidic and neutral compounds. HLB resin is available in different packages, such as the Sep-Pak-type cartridge and barrel-type columns with different volumes. Preliminary studies have confirmed the effectiveness of the HLB resin (OASIS PRIME HLB cartridge Plus Light, 100 mg; barrel-type OASIS HLB 3cc, 60 mg and OASIS HLB 6cc, 200 mg), in recovery of the [^18^F]FLT from the reaction mixture, with the product released using aqueous ethanol solution. All of these cartridges have been further evaluated in the automated mode (see next paragraph) under careful flow control, which is essential for any cartridge-based procedures.

### 2.2. Automated Synthesis of [^18^F]FLT

Having optimized reagent amounts, we moved on to the development of the automated synthesis and SPE-based purification on the GE TRACERlab FX N Pro platform. The original configuration for this module has no option to realize the black-flushing protocol that was applied in the semi-automated mode for the recovery of ^18^F-fluoride from a large QMA cartridge. Therefore, in the automated procedure a QMA-carb cartridge of 46 mg was used, allowing [^18^F]F^-^ elution with the ethanolic solution of TBAOTs in a straightforward mode [24]. Notably, for clinical radiotracers, production of the use of ethanol as a solvent is preferable, as it is relatively biocompatible. In addition, ethanol (in contrast to methanol) forms an azeotropic mixture with water, ensuring dry conditions for the following radiofluorination step. The transfer of the radiofluorination conditions established in the semi-automated mode (4.8 µmol of the PTC and 2.4 µmol of **I**) to the automated production resulted in 30% RCC, compared to 60–75% in the remote-controlled operation. The decrease in the radiofluorination efficiency can be attributed to the “non-optimal” round-shape reactor of the TRACERlab FX N Pro with larger volume (16 mL). With double the amount of reagents (9.6 µmol of the PTC and 4.8 µmol of **I**) an increase in the RCC of up to 50% was achieved. The hydrolysis step was kept as described above—heating with 1 N HCl at 100 °C for 10 min. However, the percentage of the [^18^F]FLT in the hydrolysate was only around 30% (Figure 2C), which is substantially lower than the RCC of 50% at the fluorination stage. We may assume that the part of the non-reacted [^18^F]fluoride was adsorbed on the walls of the large reaction vessel and further liberated into hydrolysate under acidic hydrolysis conditions. Finally, we pursued the purification step using three different variants of HLB cartridges. Using a Sep-Pak type cartridge (OASIS PRIME HLB cartridge Plus Light), we were faced with a problem of a high back-pressure when pushing solutions through the cartridge. Of the two barrel-type cartridges being evaluated, the 200 mg one was found to be effective, with no breakthrough of the product into waste (Table S2). The elution with 10% aqueous ethanol and passing the eluate through the Alumina N cartridge that was placed on the top of the product vial afforded radiochemically pure [^18^F]FLT in 16.0 ± 2.1% (EOB, corrected for radioactive decay) and with a synthesis time of ca. 50 min.

The radioactivity balance presented in Table 2 indicates that ca. 10% of activity (entry 2) stayed in the transfer vial for collecting irradiated [^18^O]H_2_O; these losses are unavoidable due to the TRACERlab FX N Pro module design. As for the SPE purification step, no substantial loss of the radioactivity on the cartridges was observed (6.5% on the OASIS HLB 6cc, entry 5; less than 1% on Alumina N, entry 6). RadioTLC analysis of the combined waste (46%, entry 7) showed that all the activity could be attributed to the [^18^F]fluoride (Table S2), demonstrating the high trapping efficiency of [^18^F]FLT on the HLB resin. We also observed radioactivity loss in a gaseous form, which was marked as “uncontrolled losses” (entry 9, Table 2). Comparable or slightly lower RCY (10%, corrected for decay) for the [^18^F]FLT has been recently reported [22] using a similar GE TRACERlab FX automated platform (Table 1, entry 5). The authors used 20 mg of **I** and employed a quite complex purification procedure based on four different types of SPE cartridges.

### 2.3. Quality Control

**Figure 3:**
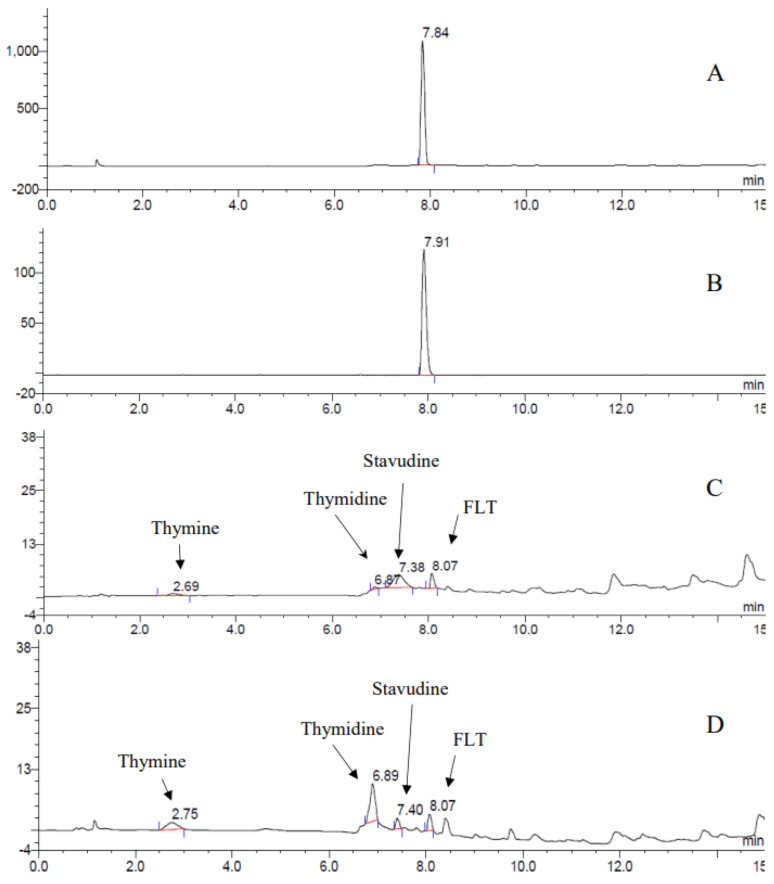
Representative radio- and ultraviolet (UV)—HPLC chromatogram obtained for the formulated [^18^F]FLT (gradient conditions, System 1, 254 nm); (**A**) UV chromatogram of FLT authentic reference; (**B**) radioHPLC chromatogram of [^18^F]FLT formulation; (**C**,**D**) UV chromatograms of two different [^18^F]FLT formulations.

The identity of the [^18^F]FLT was confirmed through an analytical radio-HPLC with a co-injection of the corresponding non-radioactive reference compound (Figure 3A). The radiochemical purity of the [^18^F]FLT was above 99%; no other radiochemical impurities were detected (Figure 3B). In addition, this parameter was checked by the radioTLC, showing >99% radiochemical purity (Figure 2D). The gradient HPLC analysis of the final formulation (Examples on Figure 3C,D) showed minimal amounts of UV-active impurities.

The amount of stavudine was as low as 0.6 ± 0.2 µg/mL, which is within the limits established by the European Pharmacopoeia Monograph (<0.1 mg/V) [23], with the volume of the final preparation being 16 mL. Also, the residual quantities of thymine—0.3 ± 0.1 µg/mL and thymidine—0.9 ± 0.2 µg/mL—were below the limits established for these impurities (<0.1 mg/V). The content of FLT in the formulated product was 1.3 ± 0.3 µg/mL, which is within the specifications of the European Pharmacopoeia monograph [23]. Molar activity of the [^18^F]FLT at the end of the synthesis (EOS) was in the range of 72–140 GBq/μmol. The final formulation of the [^18^F]FLT was proven to be sterile and pyrogen-free. All other results of the quality control analysis were within the limits specified by the European Pharmacopoeia (Table S3). The amount of residual acetonitrile was less than 400 µg/mL. Residual TBA^+^ levels in the [^18^F]FLT doses were analyzed with HPLC (system 2); no peaks with R_t_ of 4.0 min (corresponding to TBA^+^) were detected.

## 3. Experimental

### 3.1. Materials and Methods

All of the solvents and reagents were obtained from commercial suppliers and were used as-is. The precursor for the [^18^F]FLT, 3-*N*-Boc-5′-*O*-dimethoxytrityl-3′-*O*-nosyl-thymidine (**I**) was obtained from Toronto Research Chemicals (North York, ON, Canada), with a declared purity of > 95%. An authentic reference standard of the FLT (3′-deoxy-3′-fluorothymidine) was purchased from Sigma-Aldrich (St. Louis, MO, USA). The reference standards of chemical impurities—2′,3′-didehydro-3′-deoxythymidine (stavudine), thymidine and thymine were also purchased from Sigma. Anhydrous ethanol was obtained from Fluka (London, UK). Anhydrous acid-free acetonitrile (max 10 ppm H_2_O) was obtained from Kriochim (St.-Petersburg, Russia). Hydrochloric acid, sodium carbonate, methanol and tetrabutylammonium tosylate were purchased from Merck (Rahway, NJ, USA). Water from an in-house Millipore Simplicity purification system was used. All the SPE cartridges were purchased from Waters Corporation, Millford, CT, USA. Prior to use, the Oasis HLB 3cc Extraction cartridge (60 mg, Part No. WAT094226), Oasis HLB 6cc Extraction cartridge (200 mg, Part No. WAT106202) and Oasis PRIME HLB cartridge Plus Light (100 mg, Part No. 186008886) were activated by rinsing with 5 mL of ethanol following by 10 mL of water. A Sep-Pak Plus Light QMA cartridge (130 mg, WAT023525) was pretreated with 10 mL of 0.05 M NaHCO_3_ followed by 10 mL of water. The Sep-Pak Alumina N Plus cartridge (280 mg, Part No. WAT023561) was rinsed with 3 mL of water. The Sep-Pak Plus Light QMA Carbonate cartridge (46 mg, Part No. 1860044540) was used without any pretreatment.

An analytical HPLC analysis was performed using the Dionex ISC-5000 system (Dionex, Sunnyvale, CA, USA) equipped with a gradient pump, a Rheodyne type injector with a 20 μL loop, a UV absorbance detector with variable wavelength (set to 254 nm) connected in series with a radiodetector (Carrol and Ramsey Associates, CA, USA, model 105-S). The identity, radiochemical and chemical purity of the [^18^F]FLT were determined using the following HPLC conditions. System 1: X-Bridge C18 HPLC column, 150 × 4.6 mm (Waters Corporation, Millford, USA), eluent: mixture of an aqueous solution of NaH_2_PO_4_, 10 mM (pH 3 adjusted by H_3_PO_4_) with acetonitrile using gradient elution conditions, flow rate 2.0 mL/min. Gradient: 0–5.0 min 1% CH_3_CN isocratic; 5.0–15.0 min 1–95% CH_3_CN linear increase; 15.0–20.0 min 1% CH_3_CN isocratic (column equilibration). The R_t_ values for thymine, thymidine, stavudine, reference standard FLT (36 µg/mL in acetonitrile) and precursor **I** were 2.6, 6.9, 7.4, 7.9 and 14.9 min, respectively. The same HPLC conditions were used for monitoring the course of radiofluorination reaction; the R_t_ value of [^18^F] fluorinated intermediate was 15.1 min.

The residual tetrabutylammonium (TBA) level in the final product was analyzed using HPLC with the following conditions: System 2: X-Bridge C18 HPLC column, 150 × 4.6 mm (Waters Corporation, Millford, MA, USA), 20 to 90% gradient (CH_3_CN + H_2_O), flow rate 2.0 mL/min, UV 280 nm: 0–1.0 min 20% CH_3_CN; 1.0–9.0 min—from 20% to 90% CH_3_CN linear increase. The R_t_ value for TBAOH was 4.0 min.

Radio-TLC analyses were carried out on a pre-coated silica gel 60 F254 plates (Sorbfil, Lenchrom Ltd., St. Petersburg, Russia); the activity distribution was mapped using a Scan-RAM radioTLC scanner controlled by the Laura for PET chromatography software package (LabLogic, Sheffield, UK). To follow the course of the radiofluorination the crude reaction mixture was quenched by the addition of water, and an aliquot was then spotted onto a TLC plate and the plate was eluted with ethyl acetate. The R_f_ values for [^18^F]fluoride, [^18^F]fluorinated intermediate and [^18^F]FLT were 0.05, 0.55 and 0.25, respectively. The radiochemical conversion (RCC) measured by radioTLC was defined as the ratio of the product peak area to the total peak area on the TLC. The RCC value was not corrected for decay. Tests for clarity, colorlessness, residual solvents, pH value, bacterial endotoxins and sterility were performed according to the monographs on “radiopharmaceutical preparations” prescribed by the European Pharmacopoeia.

### 3.2. Production of [^18^F]Fluoride

[^18^F]Fluoride was produced via the ^18^O(p,n)^18^F nuclear reaction by irradiation of [^18^O]H_2_O (97% enrichment, Global Scientific Technologies, Sosnovyj Bor, Russia) in a silver target (1.4 mL) with 16.4 MeV protons on a PETtrace 4 cyclotron (GE Healthcare, Uppsala, Sweden). The irradiated [^18^O]H_2_O was transferred from the target using a flow of helium and loaded onto an anion exchange cartridge.

### 3.3. Remote-Controlled Radiosynthesis of [^18^F]FLT

Radiolabeling was conducted on a remote-controlled synthesis module (in-house development) with manual interventions. The module was equipped with a heating block suitable for a 5 mL reaction vessel with a screw cup (Wheaton vial). The radionuclide was transferred from the target by means of helium flow and collected in the receiving vial. Aqueous [^18^F]fluoride was loaded onto a Sep-Pak Plus Light QMA cartridge (130 mg). Residual water was removed by rinsing the cartridge with 2.5 mL of MeOH in the same direction followed by flushing with compressed air. [^18^F]Fluoride was eluted backwards relative to the loading direction using a solution of TBAOTs in 0.8 mL of MeOH; the eluate was collected into the reaction vessel. The solvent was evaporated by heating to 75 °C under gas flow and the reaction vessel was then cooled to 50 °C. The precursor **I** (1.0–4.0 mg in 1 mL of CH_3_CN) was then added to the dried residue and the reaction mixture was heated to 105 °C for 3–5 min with stirring. After slight cooling, 1 N HCl (1 mL) was added and the hydrolysis was carried out for 10 min at 100 °C with stirring. The reaction vessel was then cooled to 40 °C. The RCC was assessed by radioTLC from the aliquot of the crude mixture. The reaction mixture was partially evaporated to the volume of 0.2 mL under nitrogen flow (85 °C). After the vessel cooled to 40 °C, the crude product was then diluted with the mixture of 10 mL of H_2_O and 2.6 mL of 0.3 N NaOH with stirring. The resulting turbid solution (pH 6.5–7.5) was pushed through the Oasis HLB 3cc (60 mg), Oasis HLB 6cc (200 mg) or Oasis PRIME HLB cartridge Plus Light (100 mg), followed by rinsing with 5 mL of water. Finally, the [^18^F]FLT was eluted with 10% ethanol and further purified by passing through the Sep-Pak Alumina N Plus Light cartridge.

### 3.4. Automated Radiosynthesis of [^18^F]FLT

**Figure 4:**
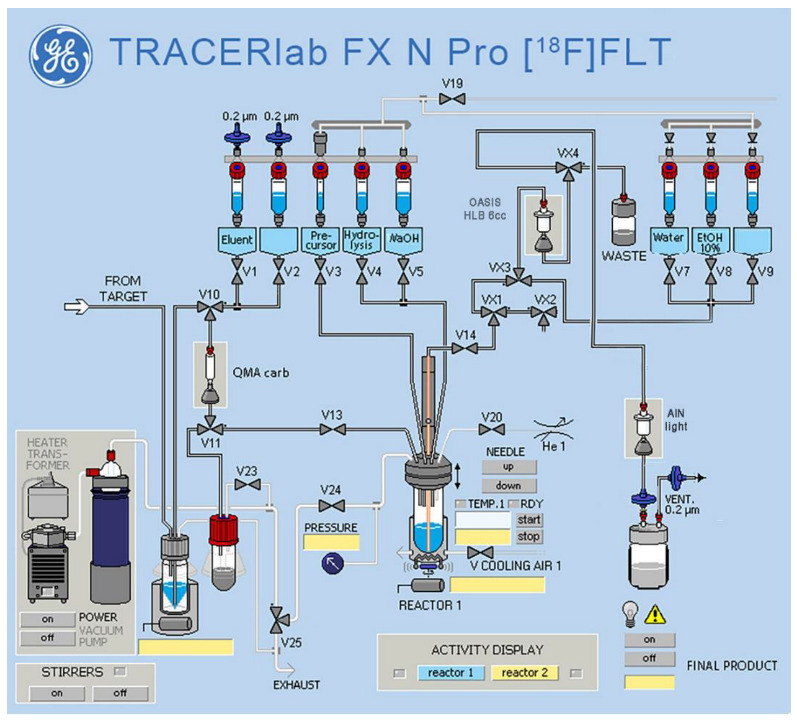
Control panel developed for the synthesis of [^18^F]FLT on the TRACERlab FX N Pro.

Radiosynthesis was performed on the TRACERlab FX N Pro (GE Healthcare, Waukesha, WI, USA). The schematic diagram and control panel as applied to the [^18^F]FLT synthesis is shown in Figure 4. Within the module reagents, transfer was achieved using nitrogen flow. Prior to the delivery of [^18^F]fluoride to the synthesis module, each vial was filled with appropriate solvents and reagents with the following reagents setup:

Vial 1—4.0 mg of TBAOTs in 2 mL of anhydrous ethanol;
Vial 2—empty;
Vial 3—4.0 mg of precursor **I** in 1 mL CH_3_CN;
Vial 4—1.0 mL 1N HCl;
Vial 5—10 mL of H_2_O and 2.6 mL of 0.3 N NaOH
Vial 7—5 mL water;
Vial 8—8 mL of 10% ethanol.

Step 1: ^18^F-fluoride preparation.

The radionuclide (5–7 GBq) was transferred from the target in a water bolus by means of helium flow and collected into the receiving vessel. The content of the vessel was pushed through the Sep-Pak Plus Light QMA Carbonate cartridge (46 mg) to recover [^18^O]H_2_O. The trapped [^18^F]fluoride was then eluted with a solution of 4.0 mg of TBAOTs in 2 mL of anhydrous ethanol into the round-bottom reaction vessel. The solvent was evaporated by heating it to 75 °C under a vacuum with stirring and the reaction vessel was cooled to 50 °C.

Step 2: Nucleophilic fluorination.

The precursor (4.0 mg in 1 mL of CH_3_CN) was then added to the dried residue and the reaction mixture was heated to 110 °C for 3 min under stirring, following by 1 min partial evaporation of CH_3_CN up to 0.2 mL under nitrogen flow (85 °C). The reaction vessel was then cooled down to 40 °C.

Step 3: Acidic hydrolysis.

To the crude product 1 N HCl (1 mL) was added and hydrolysis was carried out for 10 min at 100 °C with stirring, and the vessel was then cooled down to 40 °C.

Step 4: Neutralization.

The crude product was diluted with the mixture of 10 mL of H_2_O and 2.6 mL of 0.3 N NaOH with stirring.

Step 5. Purification and formulation.

Resulting turbid solution (pH 6.5–7.0) was pushed through the Oasis HLB 6cc, 200 mg (barrel-type) cartridge followed by 5 mL of water. Finally, the [^18^F]FLT was eluted with 8 mL of 10% ethanol, and further purified by passing through a Sep-Pak Alumina N Plus Light cartridge. The product was then collected into a vented 20 mL sterile vial pre-filled with 8 mL of buffer solution. After being passed through a 0.22 μm sterile filter (Millipore, Waters) the final product was formulated as a sterile isotonic solution with pH 7.0, containing 5% of EtOH in a total volume of 16 mL.

## 4. Conclusions

In the present work we have described an improved process for the preparation of [^18^F]FLT from the 3-*N*-Boc-5′-*O*-DMT-3′-*O*-nosyl thymidine precursor (**I**) employing TBAOTs as a non-basic phase-transfer catalyst for the preparation of reactive [^18^F]fluoride species. The synthetic procedure was developed using the GE TRACERlab FX N Pro automated synthesizer. Using TBAOTs as a catalyst, high radiofluorination efficiency was achieved with only 4 mg of the precursor used. As well as providing good yield and an easier purification route, the reduction of the precursor amount avoids the formation of radiolabeled by-products other than [^18^F]fluoride. The reduction in the amounts of chemical and radiochemical impurities allowed for the development of a fast SPE-based purification approach using a combination of OASIS HLB 6cc and Sep-Pak Alumina N Plus Light cartridges. The [^18^F]FLT was obtained in a decay-corrected radiochemical yield of (16 ± 2)% with a radiochemical purity over 99%, with the synthesis time not exceeding 52 min. The product was formulated in 16 mL of saline with 5% ethanol (*v*/*v*). The amounts of chemical impurities and residual solvents were within the limits established by the European Pharmacopoeia. The process developed compares favorably with previously reported methods due to simplicity of the automation steps, including the elimination of the azeotropic drying, and a significant reduction in the amount of the expensive precursor used. With a simple and robust SPE purification process, the method described can easily be adapted to almost any automated platform for PET radiotracers, including cassette-based modules.

## Figures and Tables

**Table 1:** Synthesis of [^18^F]FLT via radiofluorination of **I** on various automation platforms and SPE based purification; RCY—radiochemical yield, decay corrected.

I, mg	PTC	Automation Platform	SPE Purification Cartridges	RCY, %/Synthesis Time, min	Thymine Thymidine, Stavudine, μg/mL	Ref.
20	TBAHCO_3_	Modular-Lab, Eckert & Ziegler	Maxi Clean IC-H, two tC18 Sep-Pak Light, Alumina N Light	37/39	28 15 5	[16]
25	TBAHCO_3_	GE Bioscan Coincidence FDG (cassette based)	PS-H, WAX, HLB	16–39/ND	ND	[20]
40	K2.2.2/K_2_CO_3_	GE TRACERlab MX FDG (cassette based)	PS-H^+^, HLB Plus Short, Alumina Plus Long,	6.5/35	57 110 28	[28]
25	TBAHCO_3_	GE TRACERlab MX FDG (cassette based)	PS-H^+^, Oasis WAX, Oasis HLB, AluminaN	27/54	0.089–0.898 0.129 to 0.176 0.023 to 0.061	[21]
20	TBAHCO_3_	GE TRACERlab FX	Light Alumina-N, PS-H^+^, WAX plus, HLB plus	10/45	<1.2	[22]
4	TBAOTs	GE TRACERlab FX N Pro	Oasis HLB 6cc, Alumina N Plus Light	16/52	0.3 0.9 0.6	This work

**Table 2:** Activity balance (data is decay corrected, recalculated to 100%; n = 5) after synthesis [^18^F]FLT with purification on OASIS HLB 6cc + Alumina N Plus light.

Entry	Module Component	Activity %
1	Start	100
2	Transfer vial for [^18^O]H_2_O	9.0 ± 0.8
3	QMA carb 46 mg (after elution)	5.0 ± 1.3
4	Reactor	3.2 ± 1.2
5	OASIS HLB 6cc	6.5 ± 1.3
6	Alumina N Plus Light	0.9 ± 0.3
7	Combined waste	45.7 ± 5.0
8	Sterile filter + needle	2.0 ± 0.1
9	Uncontrolled losses of radioactivity	11.7 ± 2.2
10	[^18^F]FLT	16.0 ± 2.1

## Supplementary Materials

The following supporting information can be downloaded at: https://www.mdpi.com/article/10.3390/molecules27238323/s1, radio-HPLC chromatograms. Table S1: Reaction time screen. Table S2: Radiochemical yield (RCY, EOB, corrected for decay) of the [^18^F]FLT and radioTLC data for analysis of the waste obtained after passing hydrolysate through different HLB cartridges (average from three runs); Table S3. Quality control (QC) test data summary for three validation batches [23].

## Sample Availability

Samples of the compounds are not available from the authors.
