# Supplementary material for: Automated Optimized Synthesis of [18F]FLT Using Non-Basic Phase-Transfer Catalyst with Reduced Precursor Amount
Source: Molecules. 2022 Nov 29;27(23):8323. doi: 10.3390/molecules27238323 (PMC9738687; doi:10.3390/molecules27238323)
Supplement: Supplementary file 1 [file molecules-27-08323-s001.zip › molecules-2016444-supplementary.pdf]

Supporting information to

Automated optimized synthesis of [^{18}F]FLT using non-basic phase-transfer catalyst with reduced precursor amount

O. S. Fedorova, V. V. Orlovskaya, R. N. Krasikova

N.P.Bechtereva Institute of the Human Brain, 197376 St.-Petersburg, Russia;

Table S1. Reaction time screen. Conditions: 4 mg (4.8 µmol) of **I;** 2 mg (4.8 µmol) of TBAOTs, acetonitrile, 110 °C, remote-controlled procedure.

Peak assignment	The ratio of products at a given reaction time, measured by radio-TLC, %		
	5 min	10 min	20 min
[^{18}F]fluoride	46	39	30
[^{18}F]labelled side product	2	9	18
[^{18}F]fluorinated intermediate	52	52	52

Table S2. Radiochemical yield (RCY, EOB, corrected for decay) of the [^{18}F]FLT and radioTLC data for analysis of the waste obtained after passing hydrolysate through different HLB cartridges (average from three runs).

Cartridge type	Synthesis time, min	RCY, %, EOB	TLC analysis of combined waste	
			[^{18}F]F$^-$, %	[^{18}F]FLT, %
OASIS PRIME HLB Plus Light, 100 mg	57±2	15±3	92-95	5-8
OASIS HLB 3cc, 60 mg, barrel-type	52±2	14±2	60-67	33-40
OASIS HLB 6cc, 200 mg, barrel-type	52±1	16±2	100	0

Table S3. Quality control (QC) test data summary for three validation batches.

Quality control test	Ph.Eur.9.0 [23]	Validation batch 1	Validation batch 2	Validation batch 3
Radiochemical yield, corrected for decay (%)	Not applicable	15.7	17.0	16.1
Molar activity (GBq/µmol)	Not applicable	72	140	99
Radionuclidic identity (half-life, min)	105-115	110.2	111.3	110.5
Radiochemical purity,% (measured by HPLC and TLC)	>95	>99	>99	>99
pH	4.5 to 8.5	7.5	7.3	7.0

Ethanol determination (%V/V)	<10% or 2.5 g per administration	4.75	4.75	4.75
Residual solvent (acetonitrile), ppm	<400	50	68	65
Thymine (µg/ml)	Any other impurities, for each one <0.1 mg/V*	0.41	0.35	0.15
Thymidine (µg/ml)		0.71	0.90	0.99
Stavudine (µg/ml)	<0.1 mg/V*	0.54	0.33	0.65
Fluorothymidine (µg/ml)	<0.1 mg/V*	1.10	0.82	1.11
Tetrabutylammonium (µg/ml)	<2.6 mg/V*	Pass	Pass	Pass
Bacterial endotoxin (EU/ml)	<175/V*	Pass	Pass	Pass
Sterility	Sterile	Pass	Pass	Pass

*) V – maximum recommended dose in ml. The volume of the final formulation of $[^{18}F]$FLT - 16 ml.

23. Alovudine (18F) injection. 01/2014:2460. European Pharmacopoeia 9.0. 1127-1129.
